# The efficacy of initial ventilation strategy for adult immunocompromised patients with severe acute hypoxemic respiratory failure: study protocol for a multicentre randomized controlled trial (VENIM)

**DOI:** 10.1186/s12890-017-0467-6

**Published:** 2017-09-20

**Authors:** Tao Wang, Gang Liu, Kun He, Xin Lu, Xianquan Liang, Meng Wang, Rong Zhu, Zongru Li, Feng Chen, Jun Ke, Qingming Lin, Chuanyun Qian, Bo Li, Jie Wei, Jingjun Lv, Li Li, Yanxia Gao, Guofeng Wu, Xiaohong Yu, Weiqin Wei, Ying Deng, Fengping Wang, Hong Zhang, Yun Zheng, Hong Zhan, Jinli Liao, Yingping Tian, Dongqi Yao, Jingsong Zhang, Xufeng Chen, Lishan Yang, Jiali Wu, Yanfen Chai, Songtao Shou, Muming Yu, Xudong Xiang, Dongshan Zhang, Fengying Chen, Xiufeng Xie, Yong Li, Bo Wang, Wenzhong Zhang, Yongli Miao, Michael Eddleston, Jianqiang He, Yong Ma, Shengyong Xu, Yi Li, Huadong Zhu, Xuezhong Yu

**Affiliations:** 1 Emergency Department, Peking Union Medical College Hospital, No. 1 Shuaifuyuan, Dongcheng District, Beijing, China; 2 Department of Orthopedics, Peking Union Medical College Hospital, Beijing, China; 3 Department of Gastroenterology, Peking Union Medical College Hospital, Beijing, China; 4 Emergency Department, Guiyang Second People Hospital, Guiyang, China; 5 Department of Science and Technology, Beijing Tiantan Hospital, Capital Medical University, Beijing, 100050 China; 6 Department of Ultrasound, Peking Union Medical College Hospital, Beijing, China; 7 Department of Emergency, Fujian Provincial Hospital, Provincial Clinical Medical College of Fujian Medical University, Fuzhou, China; 8 Emergency Department, First Affiliated Hospital of Kunming Medical University, Kunming, China; 9 Department of Emergency, Renmin Hospital of Wuhan University, Wuhan, China; 10 Emergency Department, First Affiliated Hospital of Zhengzhou University, Zhengzhou, China; 11 Emergency Department, Affiliated Hospital of Guizhou Medical University, Guiyang, China; 12 Department of Emergency, The Second Affiliated Hospital of Harbin Medical University, Harbin, China; 13 Department of Emergency, The First Affiliated Hospital of Anhui Medical University, Hefei, China; 14 Department of Emergency Medicine, The First Affiliated Hospital of SUN Yat-sen University, Guangzhou, China; 15 Emergency Department, Second Hospital of Hebei Medical University, Shijiazhuang, China; 16 Department of Emergency, First affiliated hospital of Nanjing Medical University, Nanjing, China; 17 Department of Emergency, General Hospital of Ningxia Medical University, Yinchuan, China; 18 Emergency Department, Tianjin Medical University General Hospital, Tianjin, China; 19 Department of Emergency Medicine, Second Xiangya Hospital, Institute of Emergency Medicine and Difficult Diseases, Central South University, Changsha, China; 20 Department of Emergency, The Affiliated Hospital of Inner Mongolia Medical University, Hohhot, China; 21 Department of Emergency, Cangzhou City Center Hospital, Cangzhou, China; 22 Department of Emergency, First Hospital of Handan City, Handan, China; 23 Pharmacology, Toxicology and Therapeutics, Centre for Cardiovascular Science, University of Edinburgh, Edinburgh, UK

**Keywords:** Ventilation strategy, Noninvasive mechanical ventilation, Invasive mechanical ventilation, Acute respiratory failure, Immunocompromised patients

## Abstract

**Background:**

Acute respiratory failure (ARF) is still one of the most severe complications in immunocompromised patients. Our previous systematic review showed noninvasive mechanical ventilation (NIV) reduced mortality, length of hospitalization and ICU stay in AIDS/hematological malignancy patients with relatively less severe ARF, compared to invasive mechanical ventilation (IMV). However, this systematic review was based on 13 observational studies and the quality of evidence was low to moderate. The efficacy of NIV in more severe ARF and in patients with other causes of immunodeficiency is still unclear. We aim to determine the efficacy of the initial ventilation strategy in managing ARF in immunocompromised patients stratified by different disease severity and causes of immunodeficiency, and explore predictors for failure of NIV.

**Methods and analysis:**

The VENIM is a multicentre randomized controlled trial (RCT) comparing the effects of NIV compared with IMV in adult immunocompromised patients with severe hypoxemic ARF. Patients who meet the indications for both forms of ventilatory support will be included. Primary outcome will be 30-day all-cause mortality. Secondary outcomes will include in-hospital mortality, length of stay in hospital, improvement of oxygenation, nosocomial infections, seven-day organ failure, adverse events of intervention, et al. Subgroups with different disease severity and causes of immunodeficiency will also be analyzed.

**Discussion:**

VENIM is the first randomized controlled trial aiming at assessing the efficacy of initial ventilation strategy in treating moderate and severe acute respiratory failure in immunocompromised patients. The result of this RCT may help doctors with their ventilation decisions.

**Trial registration:**

ClinicalTrials.gov NCT02983851. Registered 2 September 2016.

## Background

Immunocompromised patients has increased subtantially over recent decades because of the epidemic of Acquired Immune Deficiency Syndrome (AIDS), common use of solid and hematologic transplantation requiring subsequent immunosuppressive therapy, better and thus greater use of iatrogenic immunosuppression, and improved survival due to better health care conditions [1]. Infection, especially pulmonary infection, is still one of the most common complications of immunosuppression [1]. Acute respiratory failure (ARF), especially when induced by sepsis, is the leading cause of ICU admission in immunocompromised patients, with a case fatality, between 30% and 90%, despite the use of antimicrobial agents and preventive measures [1–6].

Noninvasive mechanical ventilation (NIV) and invasive mechanical ventilation (IMV) are two fundamental treatment ways to provide supplemental oxygen and ventilatory support for patients with relatively severe ARF. NIV is the delivery of positive pressure ventilation via oronasal or nasal airway, a total face mask or helmet rather than endotracheal tube. The most common types of NIV are continuous positive airway pressure (CPAP) and bi-level positive airway pressure (BIPAP). By avoiding invasive intubation or tracheostomy, NIV keeps an intact upper airway and therefore retains airway defense mechanisms, which is important for immunocompromised patients who are especially vulnerable to nosocomial infections. Common complications caused by intubation such as aspiration pneumonia, ventilator-associated diseases, and trauma to adjacent organs could thus be avoided by NIV [7, 8]. NIV is now generally considered as the first-line intervention for several ARF subtypes [9–11], including chronic obstructive pulmonary disease (COPD), asthma, cardiogenic pulmonary edema and other situations [12]. However, NIV is not appropriate for all forms of ARF. In ARDS patients, for instance, the use of NIV is controversial and lacking high-quality of evidence [13–16]. Another oxygen therapy named high-flow oxygen therapy is getting more and more attention in critical care. Recent studies showed high-flow oxygen therapy obtained a comparable or even better prognosis in ARF patients [17–19]. However, it requires more advanced equipment, thus not as popularized as other conventional oxygen therapies especially in developing countries.

Two indications have been mainly considered for NIV in patients with relatively severe ARF: (1) alternative to IMV when ARF patient has contraindications to IMV, and (2) to avoid IMV when ARF patient has not yet met the criteria for intubation [20]. Generally, indications for NIV treatment include increased dyspnea (moderate to severe), tachypnea (with respiratory rate > 24 breaths per min in obstructive or >30 per min in restrictive), the use of accessory muscles, or paradoxic abdominal movements. Other indications include acute or acute on chronic ventilatory failure with hypercapnia and respiratory acidosis (PaCO_2_ > 45 mmHg and pH <7.35) and/or hypoxemia PaO_2_/FiO_2_ ratio < 200 [8]. Respiratory arrest and inability to fit the mask are considered absolute contraindications to NIV. Relative contraindications include: medically unstable (e.g. shock, uncontrolled cardiac events and uncontrolled upper gastrointestinal hemorrhage), uncooperative and agitated, uncontrolled excessive secretions, multiple organ failure, recent upper airway surgery [8].

NIV is not appropriate for all immunocompromised patients with ARF either [21, 22]. Efficacy of NIV on patients depends on disease severity, causes of immunodeficiency and forms of ARF. It’s critically important to select target patients carefully. Recently, we and our collaborators conducted a systematic review and meta-analysis [21] based on 13 observational studies (2552 patients). The study showed NIV could significantly reduce mortality, duration of hospitalization/ICU stay, and nosocomial infections mainly in less severe (reflected by the Simplified Acute Physiology Score II [23], SAPS II < 60), AIDS, and hematological malignancy subgroups, with quality of evidence from very low to moderate. However, NIV didn’t show any significant advantages over IMV in relatively more severe patients [21]. To our knowledge, evidence for other causes of immunodeficiency and different types of ARF is lacking. Early recognition of failure of NIV is also important for prognosis in these patients. Several predictors of NIV failure have been proposed to be used in immunocompromised patients, such as higher disease severity, higher respiratory rate under NIV, delayed initiation of NIV treatment, need for vasopressors or renal replacement therapy [24]. However, the cut-off point of transferring NIV to IMV in these patients is still unclear [25].

The primary goal of the present multicentre randomized controlled trial is to assess the efficacy of the initial ventilation strategy, NIV or IMV, in managing severe hypoxemic ARF in immunocompromised patients. The secondary goal of this trial is to assess the effect of NIV or IMV in patients stratified by different disease severity, causes of immunodeficiency, and presence of predictors for failure of NIV.

## Methods

### Design

The present trial is a multicentre, open-label, parallel-group randomized controlled trial of the initial **ven**tilation strategy for adult **im**munocompromised patients with severe acute hypoxemic respiratory failure (**VENIM**).

### Study setting

The VENIM trial will be conducted in the resuscitation rooms and intensive care units (ICUs) of Emergency Department in seventeen hospitals in China, including Peking Union Medical College Hospital, Fujian Provincial Hospital, The First Affiliated Hospital of Kunming Medical University, The First Affiliated Hospital of Sun Yat-sen University, Renmin Hospital of Wuhan University (Hubei General Hospital), The First Affiliated Hospital of Zhengzhou University, Jiangsu Province Hospital (The First Affiliated Hospital of Nanjing Medical University), The Second Hospital of Hebei Medical University, The First Affiliated Hospital of Anhui Medical University, The Affiliated Hospital of Guizhou Medical University, Affiliated Hospital of Inner Mongolia Medical University, General Hospital of Ningxia Medical University, Tianjin Medical University General Hospital, The Second Xiangya Hospital of Central South University, The Second Affiliated Hospital of Harbin Medical University, Cangzhou Central Hospital in Hebei province and Handan First Hospital in Hebei province. These are all tertiary level hospitals in China providing full-spectrum health services to critical patients. Referrals are from throughout China. In each hospital, staffs have been trained in the study procedures and protocols of NIV or IMV management.

### Study population

Patients will be recruited in hospitals where the staffs have considerable experience and expertise in the treatment of immunocompromised patients and application of mechanical ventilation. The inclusion and exclusion criteria are as followings (Table 1):

**Table 1:** Inclusion and exclusion criteria

Population characteristics	Inclusion criteria	Exclusion criteria
Demographic characteristics	Adult (18 years old ≤ age ≤ 80 years old) immunocompromised patients with moderate to severe ARF diagnosed within the last 72 h.	Age < 18 or >80 years old.
Diagnosis of mid to severe ARF	85 ≤ PaO_2_/FiO_2_ ≤ 170 adjusted with altitude change or clinically diagnosed with evidence of respiratory distress (intercostal recession or other assisted breathing muscle movements, polypnea >35/min or dyspnea at rest).	(1) Partial pressure of arterial carbon dioxide (PaCO_2_) > 50 mmHg or arterial pH < 7.20; (2) PaO_2_/FiO_2_ > 170(mild) or PaO_2_/FiO_2_ < 85(severe);
Diagnosis of immunocompromised status	At least one of the following: (1) Hematologic malignancy or solid tumor under chemotherapy; (2) Solid organ or stem cell transplant; (3) Long-term (>30 days) and high dose steroids (>1 mg/kg/d prednisone equivalent) usage and/or any other immunosuppressive drugs; (4) Neutropenia (defined as a neutrocyte count of <1.0 × 109/L) showing for at least 48 h.	Other causes of immunocompromise including HIV infection, etc.
Indications for intervention	Patients with severe ARF described above, in which mechanical ventilation is indicated.	Patients have been treated with NIV, IMV or high-flow oxygen therapy within 30 days. NIV is contraindicated or intubation is inevitable, which including PaO_2_/FiO_2_ < 85, respiratory arrest, hemodynamic instability, inability to fit the face mask, pneumothorax, vomiting, development of airway bleeding, inability to protect the airway, or copious respiratory secretions.
Morbidities	Mild diseases not stated in the exclusion criteria.	Acute cardiac failure; Acute-on-chronic ARF; Other severe diseases including dilated cardiomyopathy, valvular heart disease, cardiogenic pulmonary edema, implanted cardiac pacemaker, or acute coronary syndrome; systolic arterial pressure < 90 mmHg after optimal fluid therapy; history of chronic obstructive pulmonary disease (COPD) or asthma; impaired consciousness (Glasgow Coma Scale score < 8) [32]; postoperative acute respiratory failure; pregnancy or breastfeeding.
Others	Informed consent to participate in the study signed by the patient or their authorized surrogate decision maker	Lack of consent, do-not-intubate decision, and any other situations where obvious bias are expected.

*ARF* acute respiratory failure, *PaO*_*2*_ */FiO*_*2*_ ratio of the partial pressure of arterial oxygen to the fraction of inspired oxygen, *PaCO*_*2*_ partial pressure of arterial carbon dioxide, *COPD* chronic obstructive pulmonary disease, *GCS* Glasgow Coma Scale

#### Inclusion criteria

Adult (18 years old ≤ age ≤ 80 years old) immunocompromised patients with severe hypoxemic ARF diagnosed within the last 72 h, who need ventilation support, will be included in the study [26, 27].

Patients will be considered as severe ARF when the ratio of the partial pressure of arterial oxygen to the fraction of inspired oxygen (PaO_2_/FiO_2_) is between 85 and 170 [28, 29] mmHg (adjusted with [PaO_2_/FiO_2_ × (barometric pressure/760)] if altitude is higher than 1000 m [30] similarly hereinafter) or clinically diagnosed with evidence of respiratory distress (intercostal recession or other assisted breathing muscle movements, tachypnea >35/min or dyspnea at rest). PaO_2_ will be measured under oxygen therapy through a Venturi mask, and FiO_2_ will be measured by readings on the mask kit (1,117,005, Intersurgical Ltd., UK).

Patients will considered as immunocompromised when clinically diagnosed as at least one of the following:

1. Hematologic malignancy or solid tumor under chemotherapy or radiotherapy;
2. Solid organ or stem cell transplant;
3. Long-term (>30 days) and high dose steroids (>1 mg/kg/d prednisone equivalent) usage and/or any other immunosuppressive drugs [31];
4. Neutropenia (defined as a neutrocyte count of <1.0 × 10^9^/L) present for at least 48 h.

#### Exclusion criteria

Patients will be excluded from the study when:

1. Age < 18 or >80 years old;
2. Partial pressure of arterial carbon dioxide (PaCO_2_) > 50 mmHg [32] or arterial pH < 7.20;
3. PaO_2_/FiO_2_ > 170 mmHg or PaO_2_/FiO_2_ < 85 mmHg;
4. Patients have been treated with NIV, IMV or high-flow oxygen therapy within 30 days.
5. NIV is contraindicated or IMV is definitely indicated, including PaO_2_/FiO_2_ < 85 mmHg, respiratory arrest, hemodynamic instability, inability to fit the face mask, pneumothorax, vomiting, development of airway bleeding, inability to protect the airway, or copious respiratory secretions;
6. Comorbidity with acute-on-chronic ARF;
7. Comorbidity with acute cardiac failure;
8. HIV positive patients;
9. Comorbidity with other severe diseases, including dilated cardiomyopathy, valvular heart disease, cardiogenic pulmonary edema, implanted cardiac pacemaker, or acute coronary syndrome; systolic arterial pressure < 90 mmHg after optimal fluid therapy; history of chronic obstructive pulmonary disease (COPD) or asthma; impaired consciousness (Glasgow Coma Scale score < 8), [33]; postoperative acute respiratory failure; pregnancy or breastfeeding;
10. Lack of consent, do-not-intubate decision, and any other situations where obvious bias are expected.

### Sample size

The hypothesis of the present trial is that 30-day all-cause mortality in patients treated with NIV will be lower than those treated with IMV. According to previous studies, the mortality rate of ARF immunocompromised patients is from 30% to 90% [1–4]. We used the data from the Fig. 4 of our previous meta-analysis to calculate the sample size, i.e. 30-day mortality in IMV-treated patients of 125/168 (75%) [21], and 85/168 (50%) [21] in NIV-treated patients, 88 patients are needed in each group to establish non-inferiority with 90% power and a 1-sided 5% type I error rate. Assuming that 25% of patients would be lost after the enrollment, thus at least of 236 patients are required.

### Study interventions

**Fig. 1:**
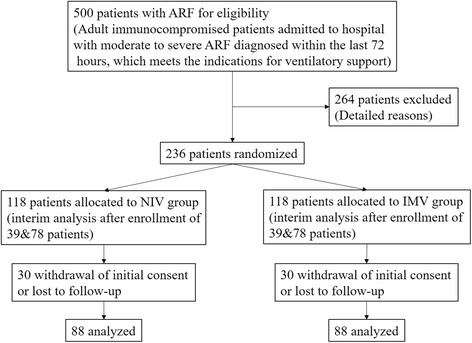
Diagram of the study protocol according to CONSORT

Patients included in the study will be randomly allocated into two groups: NIV or IMV group. The participant timeline is listed in Table 2. The study protocol and randomization arms are detailed below and in Fig. 1.

**Table 2:** Participant timeline

	Inclusion	Discharge from ICU	Day 30
Informed consent	√		
Eligibility: check inclusion and exclusion criteria	√		
Randomization	√		
Filling of case report forms		√	√
Vital status	√	√	√

**Fig. 2:**
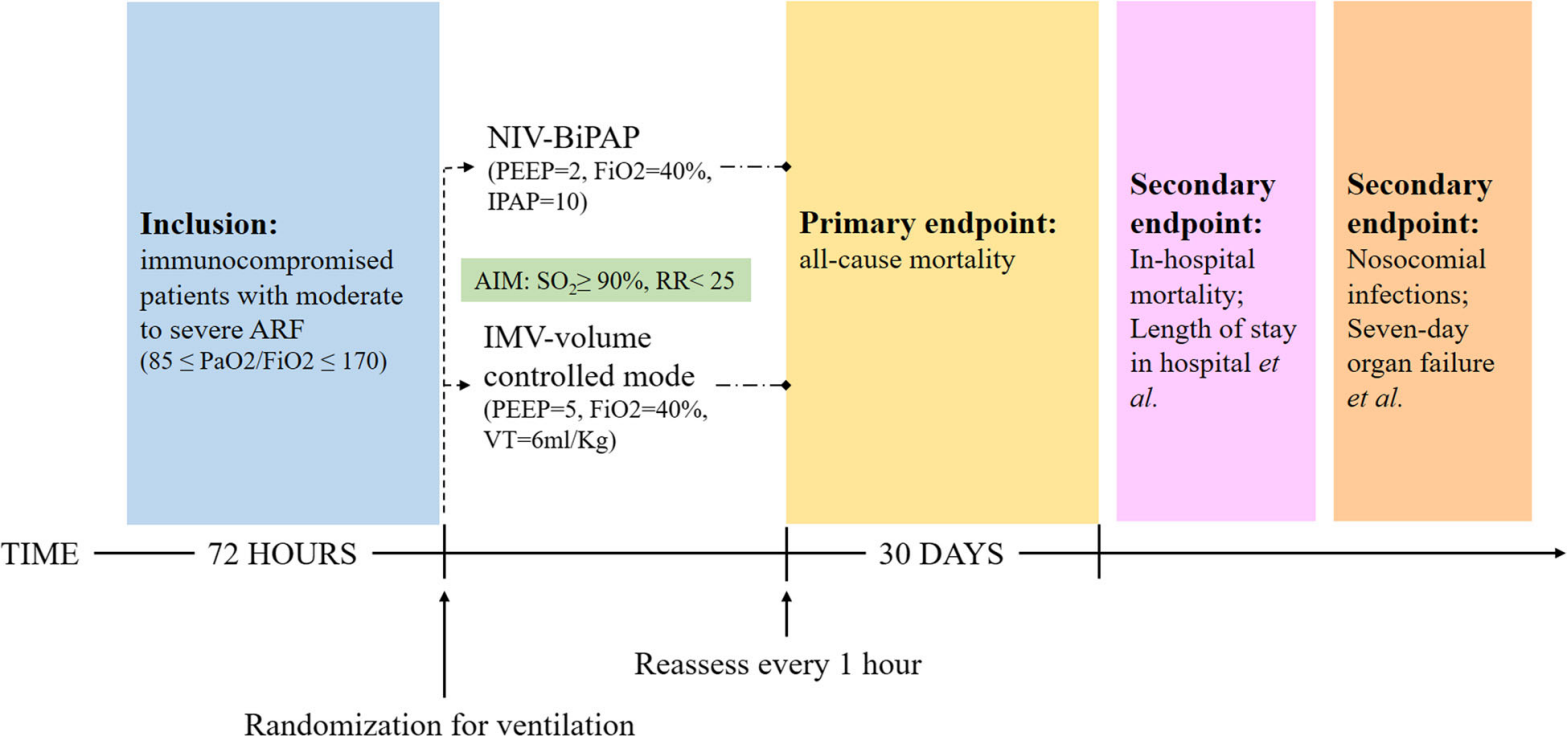
Study design of the VENIM trial. ARF, acute respiratory failure; FiO_2_, fractional inspired oxygen; IMV, invasive mechanical ventilation; IPAP, inhale positive airway pressure; NIV, non-invasive ventilation; PEEP, positive end-expiratory pressure; VT, tidal volume. BiPAP, bi-level positive airway pressure

The NIV group will receive NIV as the initial mechanical ventilation (MV) strategy, irrespective of whether IMV is used later during the following treatment. The IMV group will receive IMV as the initial MV strategy, irrespective of whether NIV is used later during the following treatment (Fig. 2).

#### Implementation of interventions

##### NIV

NIV will be delivered to the patient through a mask with built-in oxygen transport tube. The mask will be adjusted and connected to a ventilation system with oxygen concentration monitor. The initial mode will be BiPAP. In all patients, the settings of NIV will be titrated by a standard protocol: the initial settings for positive end-expiratory pressure (PEEP), FiO_2_ and Inhale Positive Airway Pressure (IPAP) will be 2 cm H_2_O, 40% and 10 cm H_2_O, respectively. PEEP levels will be adjusted in increments of 2 to 3 cm H_2_O, up to a level of 10 cm H_2_O, to obtain a FiO_2_ < 70%. All settings will be adjusted every 10 min to achieve oxygen saturation ≥ 90%, a respiratory rate < 25 breaths/min, disappearance of accessory muscle activity, and arterial blood gas tests showing PaO_2_ 60–90 mmHg, PaCO_2_ 35–45 mmHg, pH 7.35–7.45. Periods of NIV will last at least 1 h and alternate every 4 h with periods of spontaneous breathing, mouth cleaning or feeding. During 30 to 40 min’ intervals, patients breathe oxygen spontaneously while pulse oxygen saturation is continuously monitored and can be maintained above 90%.

We will continuously monitor arterial oxygen saturation, heart rate and respiratory rate in all patients. Once stabilized, patients will be reassessed every 1 h to decide the duration of NIV treatment, according to clinical assessment by the primary care team without any involvement in the research team based on the following criteria.

Criteria for NIV weaning: NIV support will be reduced progressively in accordance with clinical improvement and eventually discontinued when the patient maintains a respiratory rate < 25/min and PaO_2_ > 85 mmHg with FIO_2_ < 50% and PEEP <5 cm H_2_O and relief of respiratory distress for a period of at least 12 h.

Criteria for intubation: Patients in whom NIV fails will undergo endotracheal intubation and receive IMV. The predetermined criteria for intubation are: persistent or worsening respiratory failure at any time during the NIV treatment (high parameter settings of NIV such as FiO_2_ > 70%, PEEP > 10 cm H_2_O or IPAP > 20 cm H_2_O, failure to maintain a PaO_2_/FiO_2_ ratio > 85, respiratory rate > 35/min, PaCO_2_ > 50 mmHg or a pH < 7.30, hemodynamic instability, face mask intolerance, airway bleeding, neurological deterioration, persistent vomiting and non-compliance with NIV treatment.

##### MV

IMV will be initially performed by using the volume controlled mode, but could be adjusted to pressure controlled mode or other modes based on clinical situation such as diseases easy to develop pneumothorax, etc. The initial settings for positive end-expiratory pressure (PEEP), FiO_2_ and tidal volume (VT) will be 5 cm H_2_O, 40% and 6 ml/Kg. PEEP levels will be adjusted in increments of 2 to 3 cm H_2_O, up to a level of 20 cm H_2_O, to maintain oxygen saturation > 90%. Similar to the NIV group, the duration of IMV periods will be decided by the primary care team based on the following criteria.

Criteria and procedure for IMV weaning: IMV support will be reduced progressively in accordance with clinical improvement and discontinued if the cause for respiratory failure has been controlled, the patient maintains a respiratory rate < 25/min and PaO_2_ > 85 mmHg with FIO_2_ < 50% and PEEP <5 cm H_2_O for a period of 12 h. After a successful result of spontaneous breathing trials (including the endotracheal tubing oxygen supply, the pressure support lower than 5 cm water, or continuous positive airway pressure lower than 5 cm water for 30 min, and the Rapid Shallow Breathing Index (Tobin Index) is less than or equal to 105) has been obtained, the endotracheal tube will be extubed, and IMV support will be transferred to oxygen support via Venturi mask.

##### Concomitant medication

Supportive treatment and other necessary therapy will be given based on each patient’s clinical situation, which might include antimicrobial agents on time, diuretics, immunosuppressive agents, corticosteroids, granulocyte-colony stimulating factor, paraenteral nutrition, and crystalloids to maintain optimal volume status and to correct electrolyte abnormalities.

### Study outcomes

#### Primary endpoint

30-day all-cause mortality is the primary endpoint, which will be collected on the 30th day after patient inclusion. Information of the discharged patients will be collected via a phone call to the patient or his/her family members.

#### Secondary endpoints

1. In-hospital mortality.
2. Length of stay in hospital.
3. Length of mechanical ventilation.
4. Nosocomial infections (diagnosed according to the criteria of Centers for Diseases Control and Prevention [34]).
5. Seven-day organ failure as indicated by the Sequential Organ Failure Assessment score.
6. Adverse events of intervention (such as facial ulceration, stomach dilatation).
7. Failure of mechanical ventilation: The transfer of NIV to IMV as well as death within 30 days in NIV group will be considered as failure of NIV; Death within 30 days in IMV group will be considered as failure of IMV.)
8. Improvement of oxygenation: increase of oxygenation index by 100 compared to baseline or to 200 and above after 45 min of mechanical ventilation.
9. Continuous improvement of oxygenation: Improvement of oxygenation could be maintained till weaning or transfer of mechanical ventilation.
10. Proportion of patients intubated at 30 days (both groups).
11. Proportion of patients intubated and weaned at 3 days (both groups).
12. Ventilator-free days
13. Proportion and timing of tracheostomy.

### Study procedures

#### Recruitment

All 17 recruiting centers have substantial experience in treating immunocompromised patients with ARF. The 17 centers each admit 50 to 150 immunocompromised patients per year, 10 to 20% of whom meet VENIM inclusion criteria. Each center will screen subjects until the target population is achieved.

#### Randomization and blinding

Consecutive eligible patients will be randomly allocated in a 1:1 ratio to one of NIV or IMV. This study will include a total of 236 patients. Center 1 to Center 9 will each include 12 patients and Center 10 to Center 17 will each include 16 patients. Because the greatest common divisor of 12 and 16 is 4, we chose the block size 4. For this study, the statistician will produce computer-generated block randomization lists with SAS 9.4 (TS level 1 M2; Cary, NC, USA) stratified on the center. The randomization in blocks will be performed using opaque envelopes containing the IDs of the treatment group (NIV and IMV group). Then we will seal, shuffle the envelopes and number them in sequential order. For each eligible new patient, an envelope will be opened and then disclose the group allocation for this patient. The randomization day will be recorded as the study day zero (T0). We apply the PROBE Design [35] because ventilation cannot be blinded, and an effective sham measure is not available. Blinding to allocation of groups will be applied to the analysis.

#### Data collection and follow-up

Baseline characteristics will be recorded immediately after inclusion, including age, gender, primary diagnosis, PaO_2_/FiO_2_, causes of ARF and immunocompromised status, preexisting comorbidities, SAPS II, oxygen therapy and other treatment used before the inclusion. The information required for evaluation during study participation will be recorded daily until primary endpoint occurs or until day 30 after inclusion, whichever occurs first. Evaluations include settings of intervention, other treatment, laboratory data, evaluation of ARF, adverse events of intervention and complications developed during hospital stay. Each patient is followed until day 60 or until other endpoints occur, whichever occurs first. The following data are recorded: 30-day survival, length of hospital stay, hospital survival and adverse events of intervention.

#### Coordination and conduct of the trial

Ahead of patient recruitment, all physicians and other health-care workers in the seventeen participating hospitals will attend formal training courses on the study protocol and data collection. Data will be recorded in electronic case report form (eCRF), which is a web response system available at each study center. eCRF system is provided and managed by the Emergency Department of Peking Union Medical College Hospital. All documents required for the study are available in each center. In each participating hospital, a medical team leading by at least one senior physician will be in charge of daily patient screening and inclusion, making sure compliance with the study protocol. The principal investigators will meet with the medical teams to evaluate study progress and to discuss any problems with protocol compliance and data collection.

#### Interim analysis

Considering a large estimated sample size, two interim analyses are scheduled. Sample sizes of 176 achieve 90% power to detect a difference of 0.25 between the group proportions of 0.50 and 0.75 at a significance level (alpha) of 0.05 using a two-sided z-test with continuity correction. Assuming a dropout rate of 25%, the subjects were required to be 236. The O’Brien-Fleming spending function will be used to determine the test boundaries for 3 sequential tests (Nominal Alpha = 0.000207, 0.012025, 0.046259) [36]. One interim analysis is scheduled after enrollment of 39 patients in both group, and the second analysis after another enrollment of 39 patients in both groups. The independent data and safety monitoring committee (DSMC) consists of one biostatistician and two physicians independent of the present trial. For both interim analyses, the DSMC will monitor and assess results on day 30 mortality, failure of NIV, severe complications and seven-day organ failure as indicated by the SOFA scores. The results of the interim analyses will be kept closed unless DSMC requests premature trial termination based on these results. If the difference of the main outcome, mortality was found statistical difference between groups, then discontinuation ahead will be applied.

### Statistical analysis

All data will be analyzed based on the intention-to-treat (ITT) principle. All analysis results and findings will be reported according to CONSORT statement recommendations [37]. Descriptive statistics will be used to comparing the baseline features of the groups established by randomization. All tests will use a significance level of 0.05 and be performed using SAS 9.4 (TS level 1 M2; Cary, NC, USA).

#### Primary endpoint

30-day mortality will be reported as frequency with the 95% confidence intervals and compared between the two groups using the χ^2^ test. We will explore the interaction between treatment effect and baseline disease severity indicated by SAPS II (cut-off value 60) and PaO_2_/FiO_2_ (cut-off value 100). If such an interaction is found, we will further conduct subgroup analysis to explore a treatment effect within each subgroup. Multiple comparisons will be conducted with the Bonferroni test (error of first order 0.025). Similar analysis will be conducted between treatment effect and different causes of immunodeficiency (divided into two subgroups: a. solid tumors or hematologic malignancies, b. immunosuppressive treatment or organ transplant.).

#### Secondary endpoints

Continuous variables including length of stay in hospital, length of mechanical ventilation and seven-day organ failure will be reported as mean and standard deviation or as median and interquartile range, and compared between the two groups using Wilcoxon tests. Dichotomous variables including in-hospital mortality, nosocomial infections, adverse events of intervention, failure of mechanical ventilation and improvement of oxygenation will be reported as absolute and relative frequencies, and compared with the χ^2^ test between the two groups. Similar subgroup analysis procedures described above for primary endpoint will also be used. Kaplan-Meier estimator will be used to compare median hospital and ICU length of stay between the two groups. The event of interest and the censoring event are discharge alive and death, respectively. Intubation rate in NIV group and weaning rate in IMV group will be reported as absolute and relative frequencies. Independent variables associated with intubation rate and weaning rate will be explored with Logistic analysis model with a *p* < 0.03 in a step-by-step, backward-forward approach. In accordance with our hypothesis, the ventilatory approach and disease severity will be both forced in the model.

## Discussion

ARF is one of the most severe complications in immunocompromised patients, which still causes a relatively high mortality under the current medical status [2, 20, 38–42]. NIV is considered as an alternative to IMV in more severe patients or a means of avoiding IMV in less severe patients [20]. Patient selection is of great importance for the efficacy of NIV. On the one hand, NIV has been recommended as the first line strategy in treating ARF in immunocompromised patients by several national guidelines [26, 27]. Our recent systematic review and meta-analysis also showed NIV was a promising strategy in less severe patients (reflected by SAPS II < 60), and in patients with AIDS or hematologic malignancy [21]. On the other hand, the studies we mentioned above were all observational studies, most of which were retrospective. For other groups of immunocompromised patients not included in those studies, the efficacy of NIV is unclear. In addition, failure of NIV might delay prompt application of IMV, resulting in worse outcomes [43–46]. Therefore, a multicentre, open-label, parallel-group randomized controlled trial is needed to clarify the efficacy of initial ventilation strategy in immunocompromised patients.

High-flow oxygen therapy is indeed a potential strategy in treating ARF in immunocompromised patients and is worth more research in the future. Because difference in costs and efficacy between high-flow oxygen therapy and conventional oxygen therapies has been realized [18, 47], more and more researchers and clinicians tend to study high-flow oxygen therapy independently. Although high-flow oxygen therapy requires more advanced equipment and is not as popularized as NIV or IMV especially in developing countries, we expect the difference between the effect of high-flow oxygen therapy and NIV on acute respiratory failure in immunocompromised patients will be investigated in more future clinical trials.

## Conclusion

In conclusion, VENIM is the first randomized controlled trial aiming at assessing the initial ventilation strategy, NIV or IMV, in treating moderate and severe acute respiratory failure in immunocompromised patients, stratified by disease severity, causes of immunodeficiency, and exploring the predictors of NIV failure. The result of this RCT may help doctors with their ventilation decisions.

### Trial status

Enrollment has started on January 2017. The estimated length of inclusion time is 24 months. It is expected that recruitment will be completed by December 2018.

## Abbreviations

AIDS: Acquired immune deficiency syndrome
APACHE II: Acute Physiology and Chronic Health Evaluation II scores
ARDS/ALI: Adult respiratory distress syndrome/acute lung injury
ARF: Acute respiratory failure
BiPAP: bi-level positive airway pressure
Bi-PAP: bi­level positive airway pressure
COPD: Chronic obstructive pulmonary disease
CPAP: Continuous positive airway pressure
DSMC: Data and safety monitoring committee
eCRF: electronic case report form
FiO2: fractional inspired oxygen
GCS: Glasgow Coma Scale
ICU: Intensive care unit
IMV: Invasive mechanical ventilation
IPAP: Inhale Positive Airway Pressure
IPAP: Inhale positive airway pressure
ITT: Intention-to-treat
MV: Mechanical ventilation
NIV: Noninvasive mechanical ventilation
NIV: Non-invasive ventilation
PaCO2: Partial pressure of arterial carbon dioxide
PaO2/FiO2: ratio of the partial pressure of arterial oxygen to the fraction of inspired oxygen
PaO2/FiO2: ratio of the partial pressure of arterial oxygen to the fraction of inspired oxygen
PEEP: Positive End Expiratory Pressure
PEEP: Positive end-expiratory pressure
RCTs: Randomized controlled trials
SAPS II: Simplified Acute Physiology Scores II
SOFA: Sequential Organ Failure Assessment
VT: tidal volume
VT: tidal volume
